# Rho kinase inhibitors Y27632 and H1152 augment neurite extension in the presence of cultured Schwann cells

**DOI:** 10.1186/1749-7221-3-19

**Published:** 2008-09-25

**Authors:** Erick O Fuentes, Jost Leemhuis, G Björn Stark, Eva M Lang

**Affiliations:** 1 Department of Plastic and Hand Surgery, University of Freiburg Medical Centre, Freiburg, Germany; 2 Institute of Experimental and Clinical Pharmacology and Toxicology, Centre for Neuroscience, Freiburg, Germany

## Abstract

**Background:**

RhoA and Rho kinase inhibitors overcome the inhibition of axonal regeneration posed by central nervous system (CNS) substrates.

**Methods:**

To investigate if inhibition of the Rho pathway augments the neurite extension that naturally occurs in the peripheral nervous system (PNS) following nerve damage, dorsal root ganglion neurons and Schwann cell co-cultures were incubated with culture medium, C3 fusion toxin, and the Rho kinase (ROCK) inhibitors Y27632 and H1152. The longest neurite per neuron were measured and compared. Incubation with Y27632 and H1152 resulted in significantly longer neurites than controls when the neurons were in contact with Schwann cells. When separated by a porous P.E.T. membrane, only the group incubated with H1152 developed significantly longer neurites. This work demonstrates that Rho kinase inhibition augments neurite elongation in the presence of contact with a PNS-like substrate.

## Background

The CNS is an environment normally hostile to nerve regeneration due to the presence of axonal inhibitory substrates like chondroitin sulphate proteoglycans (CSPGs) – present in both the glial scar and in myelin – NOGO and myelin associated glycopeptide (MAG) [1]. These substances inhibit axonal regeneration by activating on RhoA, a member of the Rho GTPase family. Active RhoA causes the retraction of growth cones by increasing the net phosphorylation of the myosin regulatory light chain. It also activates Rho kinase (ROCK) which directly phosphorylates the regulatory light chain of the major cytoplasmic myosin, myosin II, increasing its actin-activated ATPase and thus contractility [2] resulting in growth cone collapse and retraction [3,4]. RhoA activity is increased following CNS injury [5] further augmenting the inhibition of axonal regeneration that is already present. It is known that this effect is overcome by the RhoA specific inhibitor C3 transferase and the ROCK-specific inhibitor Y27632 [6-8,1]. The p75 nerve growth factor (p75NTR) plays an important role in the axon and neurite extension through modulation of the RhoA pathway. In the unbound state, the p75NTR constitutively activates RhoA. When neurotrophin binds to the p75NTR, RhoA activation is switched off [9-11]. The CNS inhibitory substrates such as NOGO mediate their effect by binding to the p75NTR however, this binding causes the activation of RhoA and hence the inhibition of axonal regeneration [12,7].

In contrast to the CNS, the peripheral nervous system (PNS) allows nerve regeneration to occur following nerve injury such as axotomy or crush injury. This is assisted by Schwann cells (SC), which provide neurons with adhesion molecules and a myriad of neutrophins to support neurite and axonal growth. Little is known of the role that Rho GTPases play in peripheral nerve regeneration. Whilst RhoA is present and expressed in peripheral nerve axons and SC [13], recent work suggests that RhoA activity is not increased in SC following PNS injury [14]. Rho has also been shown to play a role in the process of PNS myelination [15,16]. and SC migration [17]. There is however, sparse evidence showing that axonal regeneration or neurite elongation are enhanced by the inhibition of RhoA or ROCK in the PNS hence, this work aimed to measure the effect of Rho and ROCK inhibition on neurite extension of neurons on a PNS like environment.

## Materials and methods

### RhoA and ROCK inhibitors

The C3 fusion toxin (C3 FT), a chimeric protein consisting of the Clostridium limosum toxin C3 and the N-terminal adaptor domain of Clostridium botulinum C2I, which interacts with the binding/transport component C2II of the C2 toxin. In this construct, the C2II protein acts as a pore forming protein which allows the efficient delivery of the C3 protein into target cells [18]. C3 FT/C2II toxin was used at 10 ng/ml:20 ng/ml concentration. (C3 FT and C2II proteins were kindly donated by Dr. K Aktories, Institute of Experimental and Clinical Pharmacology and Toxicology, Freiburg, Germany).

Y27632, is a well established inhibitor of ROCK in a variety of systems. This pyridine derivative is the oldest synthesised and reported specific inhibitor of Rho-kinase family enzymes. Y27632 inhibits ROCK activity by competitive binding with ATP to the catalytic domain. Y27632 is reported to have a specificity 100 times greater for ROCK than for protein kinase A, protein kinase C, or myosin light chain kinase, as well as over 20 times greater than that for two other downstream Rho effectors, citron kinase and protein kinase N [19,20]. Y27632 (Calbiochem, USA) was dissolved in 1 ml of distilled water, smaller aliquots using culture medium were made and a final concentration of 10 μM was used.

The newer H1152 is a more specific, stronger and membrane-permeable inhibitor of ROCK with a Ki value of 1.6 nM. It is a poor inhibitor of the serine/threonine kinases, PKA, PKC and MLCK. The Ki values of H1152 for these kinases are about 390, 5800 and 6300 times higher than for Rho-kinases, respectively [21-23]. H1152 (Calbiochem, USA) was dissolved in distilled water and used in a concentration of 100 nM.

### Cell Cultures

Schwann cell cultures were prepared from sciatic nerves of 2 to 3-day-old Wistar rats. These were surplus animals from the animal breeding program belonging to the Faculty of Veterinary Science of the University of Freiburg. The animals were housed and handled in accordance to the local animal ethics committee rules. The rats were given a lethal dose of CO_2_, the sciatic nerves excised and placed into ice cooled DMEM (GibcoBRL Life Technologies, Germany). The epineurium was removed, the nerves then cut into small blocks and digested in 2 ml of DMEM with 0.25% trypsin (Sigma, Germany) and 0.1% collagenase A (Roche Diagnostics, Germany) for 45 min at 37°C. The nerve pieces were mechanically dissociated using a fire polished Pasteur pipette and the cells collected by centrifugation and resuspended in DMEM with 1000 IU/ml penicillin, 1000 mg/ml streptomycin, and 10% FCS. The SC were plated on 25 ml culture bottles, the medium was changed after 24 h and cytosine-arabinoside 1 μM/ml (Sigma Aldrich, Germany) added. After 48 h, the cells were passaged under microscope control using 1 ml of trypsin/EDTA (PAA Laboratories, Austria). The trypsin activity was stopped with serum containing medium and the cells gathered by centrifugation at 1000 rpm for 5 min at 4°C. The cells were resuspended in 5 ml of medium, plated onto a new 25 ml culture bottle and the mitogen activator forskolin 1 μM/ml (Calbiochem, USA) added to the medium. Primary SC cultures became 80% confluent after 14 to 20 days. The medium was changed every 7 days.

### Dorsal Root Ganglion Neurons

Cultures of dorsal root ganglions were harvested from the same rats and DRG neuron cultures established using the method described by Seilheimer [24]. In short, the spinal columns from 10 rats were excised and washed in ice cooled PBS (Biochrom, Germany). Under a dissecting microscope the vertebral lamini were removed and 20 to 25 DRG were dissected from each animal and placed in ice cooled DMEM. The neurons were dissociated in 0.25% trypsin and 0.1% collagenase in 2 ml of DMEM for 45 min at 37°C followed by mechanical dissociation and centrifugation. The cells were resuspended and passed through a 50 μm pore size cell strainer. After 5 min of gentle centrifugation they were transferred onto a cushion of 32% Percoll (Sigma Aldrich, Germany) and centrifuged for 10 min at 4°C and 600 rpm. Using a cell counting chamber the concentration of neurons was estimated and an appropriate dilution and aliquots of the suspension set aside for plating onto the coverslips.

### Neuron enriched cultures

150–200 neurons were plated onto rat-tail collagen coated coverslips. The cultures were kept overnight in DMEM medium with 1% penicillin and 10% fetal calf serum (FCS) and supplemented 10 ng/ml of nerve growth factor (NGF) (Sigma, Germany). The medium was subsequently replaced, NGF omitted, and C3 FT, Y27632 and H1152 (Merk/Calbiochem, USA) at concentrations of 10 ng/ml, 10 μM and 100 nM respectively, added to the medium and the culture incubated for 8 h. The control group was incubated only in medium without NGF. At the end of the incubation, the cells were fixed with 4% paraformaldehyde in cytosol stabilization buffer (CSB).

### Co-culture experiments

DRG neurons and Schwann cells were put in co-culture in two distinct ways. For direct neuron-SC contact, 30,000 Schwann Cells were plated onto 10 mm diameter poly-L-lysine (Sigma-Aldrich, Germany) coated glass coverslips and kept for 48 h in DMEM with 10% FCS, 150–200 dissociated DRG neurons were seeded directly onto this confluent SC culture. Alternatively, the same number of SC were plated onto a chamber insert containing a P.E.T. membrane with 1 μm pores (Falcon, Denmark). 48 h later, 150–200 neurons were seeded onto coverslips coated with rat tail collagen and the aforementioned chambers inserts were placed in the same culture well sharing the same medium.

The co-cultures were allowed to stabilize for 4 h after which C3 Fusion Toxin, Y27632 and H1152 were added to the medium at a concentration of 10 ng/ml, 10 uM and 100 nM respectively. They were then incubated for a further 8 hours. The length of the longest or more dominant neurite seen to arise from a neuronal cell body was measured. Neurons were excluded from the analysis if; a) the neurites were in contact with neurites or the cell body of other neurons, b) had highly branched neurites that made it impossible to determine the presence of a dominant neurite. The neurons plated onto coverslips not containing SC invariably had some contaminating SC. In this case, only the neurites of neurons not in contact with SC were included in the analysis. Two coverslips were used in each experiment for each substance. Each experiment was repeated 4 times.

### Immunohistochemistry

**Figure 1:**
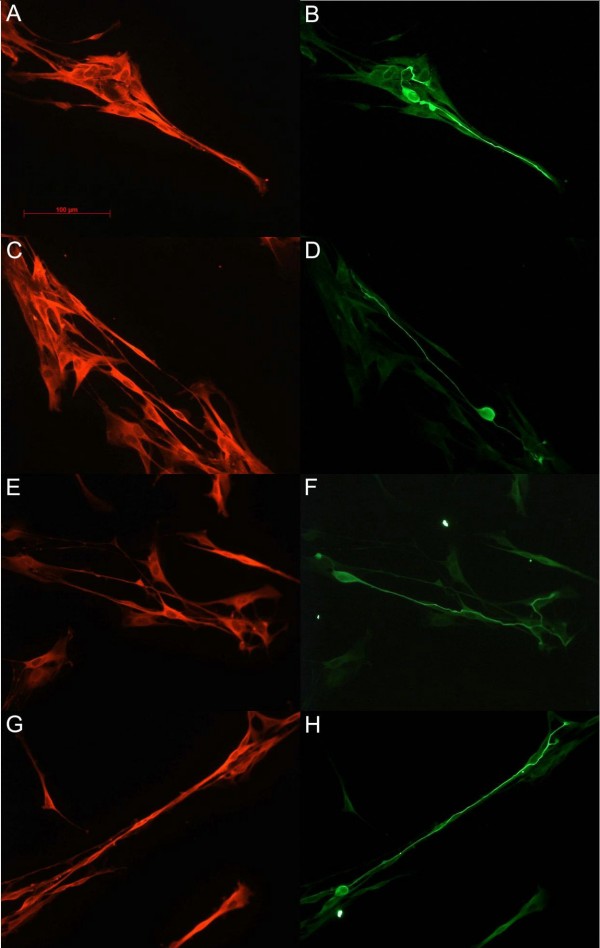
**Double immuno-fluoresence of Schwann cell and DRG neuron co-cultures seen under ×40 objective. **Images A (control), C (C3 Toxin-treated), E (Y27632-treated), and G (H1152-treated) show the Schwann cell-specific S100 stain. Images B, D, F, and H are the corresponding Beta tubulin stains of A, C, E and G respectively.

The method used for immunohistochemistry was adapted from that described by Henle [25]. In short, coverslips containing co-cultures were fixed for 12 min in 4% paraformaldehyde (PFA) in cytosol stabilization buffer (CSB), followed by 2 min in 0.1% triton-x in CSB. The S100 rabbit anti-rat primary antibody (DAKO, Denmark) and primary mouse anti-βtubulin III (Sigma Aldrich, Germany) were added at a concentration of 1:200 and 1:650 v/v respectively in 1% goat serum in PBS and incubated for 1 h. After rinsing with PBS the coverslips were incubated with the Cy3-conjugated goat anti-rabbit (Dinova, Germany) and Alexa-488 coupled goat anti-mouse secondary antibodies for 1 h in the dark at room temperature. The coverslips were mounted onto glass slides using Prolong Gold anti-fade (Molecular Probes, Neatherlands) mounting medium (Figure. 1).

### Microscopy and image and data analysis

Fixed co-culture coverslips were loaded onto glass slides and looked at using the ×40 magnification under an Axioplan 2 microscope with epifluorescence mounted with a digital Axiocam camera (Carl Zeiss, Germany). Cells of interest were photographed using the Axiovision 3.01 program (Carl Zeiss Vision, Germany). The images were saved and later analysed using the built-in measuring function "length", available in the Metamorph Version 6.1r4 image analysis software (Universal Imaging Corp. USA). This function automatically measured the overall length after drawing a box around the selected neurite [26].

The data obtained from the neurite measurements were analysed with Microsoft Excel 2002 (Microsoft Corporation) and SPSS 12.0.1 for Windows (SPSS Inc.). The two-tailed T-test was used to compare of the proportion of neurons that responded to the different stimuli. Neurite lengths were compared using one-way ANOVA with Tamhane's post hoc test.

## Results

### C3 fusion toxin and the Rho kinase inhibitors Y27632 and H1152 increase neurite length in neuron-enriched cultures

**Figure 2:**
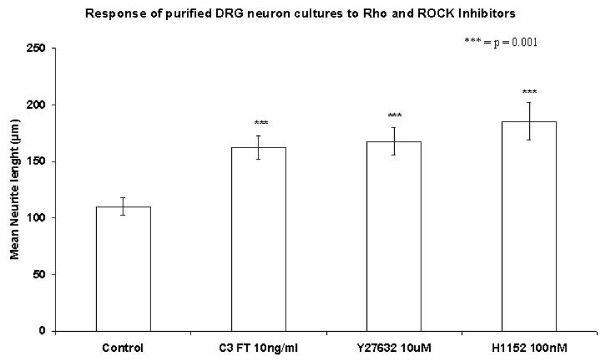
Neurite length of purified DRG neurons after an overnight incubation with 10 ng/ml NGF followed by 8 h incubation with C3 FT, Y27632 and H1152.

Dissociated DRG neuron cultures incubated overnight with 10 ng/ml of NGF followed by an 8 h incubation with C3 fusion toxin and the ROCK inhibitors Y27632 and H1152 showed an increase in the average length of their longest neurites when compared to the control group (medium only). The average maximal neurite lengths were in the controls 110.1 μm (SE ± 7.8) in the C3FT group 162.4 μm (SE ± 10.4), in the Y27632 group 167.8 μm (SE ± 12.3), and 185.4 μm (SE ± 16.6) in the H1152 group. All groups showed significantly longer neurites than the control group (p < 0.001). There was no significant difference between the three treatment groups (Figure. 2).

### Effects of Rho and ROCK inhibition on SC morphology

The effect of Rho kinase inhibition on Schwann cell morphology was previously described [15]. The most notable change in SC morphology observed in this study was caused by Y27632 and to a lesser extent by H1152. These SC showed narrower and longer spindles with a more triangular cell body. There was no obvious morphologic effect of C3 FT on Schwann cells.

### Inhibition of RhoA and ROCK has no effects on number of neurons that extend neurites

The proportion of neurite-bearing neurons (i.e. DRG neurons with neurite sprouts) was estimated by counting all neurons with neurites present in one half of the coverslips and dividing by the total number of neurons present in that section of the coverslip. The proportion of neurite-bearing DRG neurons in direct contact with SC was 44.5% in the control group, 38.6% in the C3 FT group, 52.4% in the Y27632 group and 52.0% in the H1152 group. The proportion of neurite-bearing neurons separated form SC by the presence of the separation membrane were 10.3%, 7.2%, 10.3%, 13.1% respectively. In both experimental settings, there were no statistically significant differences between stimuli and controls.

**Figure 3:**
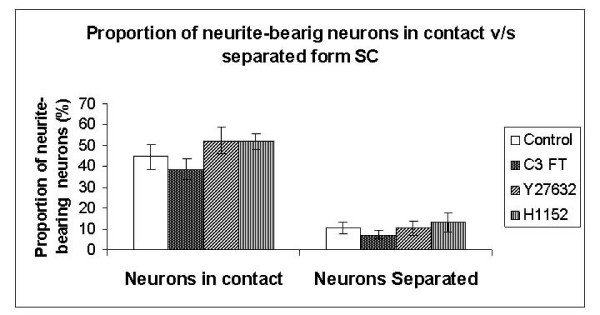
**Histogram comparing the proportion of neurite-bearing neurons under the two co-culture conditions.** As expected, there was a highly significant difference between the neurons in contact vs separated from Schwann cells for the same test stimuli (p ≤ 0.005) however, there were no significant differences between the different stimuli under the same culture conditions.

The proportion of neurite-bearing neurons in contact with Schwann cells were clearly higher in all groups when compared to neurons separated from SC by the 1 μm pore-size P.E.T. membrane with permeable membrane (p < 0.001, Figure. 3).

### Y27632 and H1152 promote neurite extension in neurons which are in contact with Schwann cells

**Figure 4:**
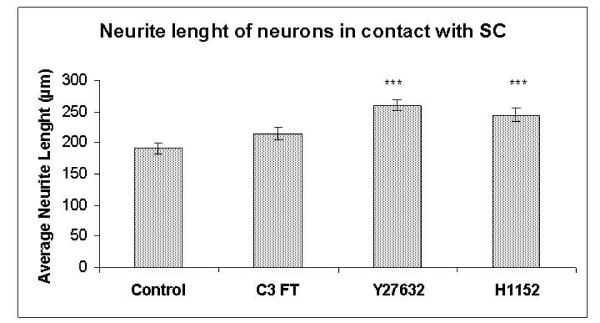
**Histogram comparing neurite lengths of neurons in contact with Schwann cells after incubation with C3 FT, Y27632 and H1152.** *** p ≤ 0.001.

Next the length of the longest neurite of the neurons, which were in direct contact with SC were studied. To this end 144 control, 141 C3 FT, 138 Y27632 and 137 H1152-treated neurons were analysed. Interestingly, neurons in the C3 FT, Y27632 and H1152 groups had on average longer neurites than in the control group (Figure. 4). The average neurite lengths obtained were 190.4 μm (SE ± 8.3) in the controls, 213.8 μm (SE ± 9.5) in the C3 FT group, 259.7 μm (SE ± 9.1) in the Y27632 group and 244.4 μm (SE ± 11.4) in the H1152 group. However, only the neurite lengths in the Y27632 and H1152 groups were significantly longer than controls (p < 0.001 and p = 0.001 respectively). There was no significant difference between the C3 FT, Y27632 and H1152 groups (Figure. 4).

**Figure 5:**
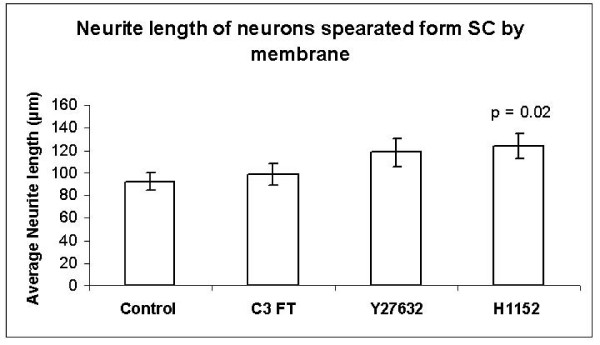
Histogram comparing neurite lengths of neurons separated from Schwann cells by a porous P.E.T. membrane after incubation with C3 FT, Y27632 and H1152.

When using the separation chamber to prevent SC-neuron contact, the longest neurites from 60 control, 45 C3 FT, 58 Y27632 and 38 H1152-treated neurons were included in the analysis. The average neurite lengths for the separated neurons were for 92.2 μm (SE ± 8.0) in the Control group, 98.8 μm (SE ± 10.1) in the C3 FT group, 118.0 μm (SE ± 12.6) in the Y27632 group and 123.5 μm (SE ± 10.9) in the H1152 group. Although the average neurite lengths were longer in the C3 FT, Y27632 and H1152 groups, only the H1152 treated group was significantly longer than the control group (p = 0.02, see Figure. 5).

**Figure 6:**
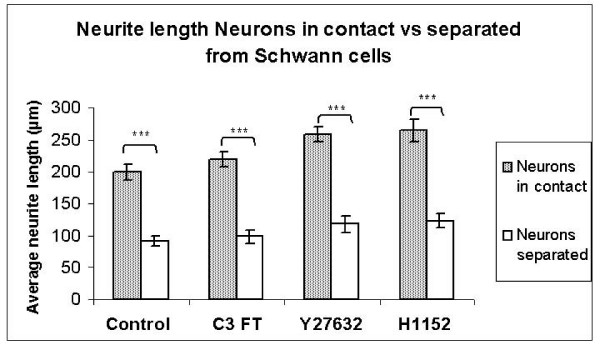
**Histogram comparing the neurite lengths for neurons in contact with vs separated from Schwannn cells after treatment with C3 FT, Y27632 and H1152.** *** p ≤ 0.001.

The neurite lengths of the neurons in contact with SC were significantly longer than the separated neurons (see Figure. 6). The p values were all < 0.001.

## Discussion

Axonal or neurite elongation following the inhibition of RhoA and Rho Kinase is well described. For instance, recombinant constitutively active RhoA transfected into PC12 cells causes suppression of neurite extension, which is reversed by C3 whilst transfection of dominant negative Rho results in increased axonal and neurite length [27]. The use of both C3 transferase and the Rho kinase inhibitors Y27632 to promote neurite extension on foetal neurons has been demonstrated by various authors [27-29]. Other authors have reported increases in axonal length form DRG explants following incubation with C3 and Y27632 [1]. We observed a similar response in neonatal DRG neuron enriched culture experiments, where neurite length was increased by C3 FT, H26732 and the newer and more specific Rho kinase inhibitor H1152.

Rho and ROCK inhibitors overcome the inhibitory effect that CNS substrates have on axonal regeneration [30]. It has been shown that substances like chondroitin sulphate proteoglycans (CSPGs), NOGO and myelin associated glycopeptide (MAG) [31,32]. mediate their inhibitory effect on axonal regeneration by the activation of the RhoA pathway [1] by binding to the NOGO receptor which interacts with the p75NTR receptor which, in turn constitutively activates Rho [33,7,34,28]. This work aimed to ascertain if the regenerative properties of Rho and ROCK inhibitors also apply in the peripheral nervous system model when the aforementioned inhibitory substrates are not present i.e. in the *in vitro *non-myelinating SC culture.

Cultured SC have properties similar to the SC in injured nerves following Wallerian regeneration [35,36]. Importantly, SC synthesise and secrete various neurotrophins including nerve growth factor (NGF), neurotrophin 3 (NT-3), brain derived neurotrophic factor (BDNF) and ciliary neurotrophic factor (CNTF) [37,38]. Since the p75NTR binds all neurotrophins with a similar affinity [39,40], it can be expect that all these neurotrophins will cause the inactivation of RhoA by their binding to the p75NGFR.

As a result, the null hypothesis ie. that additional extrinsic inhibition of the Rho/ROCK pathway would have no added effect on axonal/neurite elongation in our PNS model was formulated. This hypothesis is supported by the work of Geheler *et al *who demonstrated that neurons treated with C3 showed no further increase in filopodial length when co-incubated with BNDF. This effect was shown to be independent of the Trk pathway since blockade with K252a at the same time that RhoA inhibition was induced by neurotropin binding to the p75 receptor, resulted in filopodial extension of the same magnitude as when the p75 receptor was stimulated with an intact Trk pathway [11]. In contrast to previous studies where DRG explants were incubated in medium containing NGF, NT-3 or BDNF [30,11]., the co-culture experiments in this study did not use extrinsic growth factors. Rather, they relied on the trophic substances secreted by the SC to ensure the survival of the cultured neurons. This makes it possible to observe if these Rho kinase inhibitors also influence the number of neurons that extend neurites in the co-culture setting.

The proportion of neurons that sprouted neurites following a challenge with the experimental substances were not significantly different to the control group or to each other in the two co-culture conditions tested in this study. If we consider neurite sprouting/bearing as a surrogate measure of neuronal survival or viability, then it is clear that inhibition of the RhoA pathway does not improve nor worsen neuronal survival. This agrees with previous findings by other authors where RhoA inhibition resulted in motor neuron apoptosis during development but it did not seem to affect sympathetic or DRG neuron survival [41] with the essential difference that this study used neonatal rather than embryonic cells. Our experiments showed significant differences between the number of neurons-bearing neurites in the two co-culture conditions. Although equal numbers of SC were seeded onto the coverslips (neurons in contact with SC) and separation chambers (neurons separated from SC), a significantly greater proportion of neurons sprouted neurites when in contact with SC. Similarly, the neurite lengths of neurons in contact with SC were significantly greater in comparison to their counterparts separated from SC by the permeable membrane. These results would suggest that the number of SC used did not provide enough trophic support via diffusible factors to adequately maintain the viability of the seeded neurons in the separation chamber experiment. This result was anticipated and is consistent with the literature where it is known that neuron-SC contact improves neuron survival and axonal regeneration [42,43,24,37]. Adhesion molecules present and SC such as L1 have been shown to mediate a trophic effect that ensures axonal survival [44]. Similarly, laminin which is a constituent of the Schwann cell basal lamina, has also been demonstrated to override the inhibitory effects of PNS and CNS derived inhibitors of neurite growth [45]. Hence it can be concluded that the discrepancy in results between the two co-culture conditions is due to a fundamental difference in the strength of the trophic support provided by the Schwann cells rather than a lack of effect from the RhoA and ROCK inhibitors used. It may be argued that the results observed in the neurons in contact with SC more closely resemble peripheral nerve injury *in vivo*.

Our null hypothesis was disproved. The co-culture experiments showed that both Y27632 and H1152 highly significantly increased the lengths of neurites when the neurons were in contact with SC. Neurons separated from SC also showed an increase in neurite length when incubated with C3 FT, Y26732 and H1152 however, this increase was only significant in the H1152 group. Unfortunately, technical difficulties with this experiment, namely the extremely low number of neurites extended by the neurons (see above), did not allow the collection of a greater number of observations for each group. It may well be that with a greater number of observations, the effects of Y27632 and C3 FT would become significant. Another strange observation was that, although C3 transferase showed a small increase in neurite length, this was not significant in any of the co-culture experiments. It is difficult to explain why C3 transferase had a significant effect in the neuron enriched culture and yet no effect in the co-culture experiments. One possibility is that either the C3 or the C2II proteins were denatured or had expired and thus no longer effective at the time of the co-culture experiments however, a fresh batch of these proteins was used in each experiment. Another possible explanation, although less likely, is that the toxin complex was preferentially taken up by the SC thus decreasing the effective concentration delivered to the neurons. If this were the case, one would expect the SC to undergo a morphological change. However, in this study, the SC did not show a significant morphological change with C3. Nevertheless, given the increases in neurite lengths observed in these experiments, it can be expected that ROCK inhibitors would increase axonal extension *in vivo*.

Whilst a recent study using the sciatic nerve crush injury model showed that peripheral nerve regeneration is enhanced by ROCK inhibition, it did not describe a direct measure of increased axonal or neurite length [14], rather they used surrogate measures such as compound muscle action potential and motor nerve conduction studies. For the first time, we show that incubation of neurons with ROCK inhibitors increase neurite length while neurons are in the presence of an environment similar to that of the PNS following Wallerian degeneration. In this state, Schwann cells shed their myelin and the myelin is then phagocytosed [46,47]. This leaves connective tissue tubes lined with proliferating Schwann cells that form linear bands within the endoneurial sheath, known as Bands of Bungner, which are important in guiding regenerating axons across the injury site and into the distal nerve stump [36]. However, given that neonatal neurons have a higher intrinsic growth capacity [48] it is uncertain if these observations would hold true when using an adult animal model.

Taken together, these results suggest that inhibition of RhoA and ROCK in peripheral sensory neurons in the presence of cultured Schwann cells, will result in axonal or neurite extension. A reasonable explanation is that given that RhoA and ROCK inhibition favour growth cone extension by limiting growth cone collapse and retraction [2,1], contact with Schwann cells (in their unmyelinating state) favours further extension because of the presence of adhesions molecules on their surface, which provide greater stabilisation of the growth cone and in turn allows further elongation. However, the short duration of these experiments pose further questions. For instance, it was observed that the ROCK inhibitors Y27632 and H1152 altered the morphology of the SC. These changes were similar to those observed by Melendez *et al *[15]. They also described a shortening of the myelin segments provided by myelinating SC and detachment of SC for the culture substrate after 48 hours or incubation with C3 and Y27632. The latter is in keeping with the fact that Rho GTPases are involved in the regulation of cell adhesion to substratum and in cell to cell adhesion [49]. How these observations would translate to *in vivo* peripheral nerve regeneration in the presence of Rho and Rho kinase inhibition remain undescribed.

## Conclusion

In conclusion, we propose that inhibition of the RhoA pathway in the peripheral nerve model, results in increased neurite or axonal length. This may be due to Schwann cells promoting the stabilisation of the growth cone and thus, shifting the balance in the dynamics of growth cones in favour of axonal elongation. Further work using an *in vivo* model is warranted to gain further knowledge of the effects of RhoA and ROCK inhibitors on the functional recovery afterperipheral nerve injury.

## List of abbreviations used

BDNF: brain derived neurotrophic factor; CNTF: ciliary neurotrophic factor; CNS: central nervous system; C3 FT: C3 fusion toxin; CSPGs: chondroitin sulphate proteoglycans; CSB: cystosol stabilization buffer; DRG: dorsal root ganglion; FCS: fetal calf serum; MAG: myelin associated glycopeptide; NGF: Nerve growth factor; NT-3: neurotrophin 3; PBS: phosphate buffered saline; PFA: paraformaldehyde; PNS: peripheral nervous system; ROCK: Rho kinase.

## Competing interests

The authors declare that they have no competing interests.

## Authors' contributions

EF designed the experimental protocols, carried out the experimental work, microscopy, data analysis, and prepared the manuscript. EL helped to design the experimental protocols, assisted in data analysis and interpretation and preparation of the manuscript. JL intellectually contributed to the experimental design. GBS helped in correction the manuscript. All Authors have read and approved the final manuscript.
